# Do delusions have and give meaning?

**DOI:** 10.1007/s11097-021-09764-9

**Published:** 2021-08-23

**Authors:** Rosa Ritunnano, Lisa Bortolotti

**Affiliations:** 1 Institute for Mental Health, University of Birmingham, Edgbaston, Birmingham, B15 2TT UK; 2 Philosophy Department and Institute for Mental Health, University of Birmingham, Edgbaston, Birmingham, B15 2TT UK

**Keywords:** Delusions, Meaning, Meaningfulness, Phenomenology, Cognitive psychology, Sense of coherence, Creativity

## Abstract

Delusions are often portrayed as paradigmatic instances of incomprehensibility and meaninglessness. Here we investigate the relationship between delusions and meaning from a philosophical perspective, integrating arguments and evidence from cognitive psychology and phenomenological psychopathology. We review some of the empirical and philosophical literature relevant to two claims about delusions and meaning: (1) delusions are meaningful, despite being described as irrational and implausible beliefs; (2) some delusions can also enhance the sense that one’s life is meaningful, supporting agency and creativity in some circumstances. Delusions are not incomprehensible representations of reality. Rather, they can help make sense of one’s unusual experiences and in some circumstances even support one’s endeavours, albeit temporarily and imperfectly. Acknowledging that delusions have meaning and can also give meaning to people’s lives has implications for our understanding of psychotic symptoms and for addressing the stigma associated with psychiatric conditions.

## Introduction

In this paper, we focus on the phenomenon of clinical delusion and address two issues from the perspectives of cognitive psychology and phenomenology: (1) Are delusions meaningful? (2) Can delusions contribute to the sense that one’s life is meaningful? Clinical delusions are unusual beliefs that are thought to be symptomatic of mental disorders. In particular, here we consider delusions reported by people who have attracted a diagnosis of schizophrenia or psychotic illnesses.

Clinical delusions are regarded as paradigmatic instances of pathological beliefs, and some philosophers have even challenged their doxastic status, arguing that delusions may sound like beliefs but are not genuine instances of belief, because they do not share the functional profile of beliefs. For instance, delusions are to some extent “fixed”, whereas we expect beliefs to be responsive to counterevidence; and delusions are not always manifest in behaviour, whereas we wexpect beliefs to drive action (for a review of anti-doxastic arguments, see Bortolotti & Miyazono, 2015). Other philosophers have claimed that delusions cannot be belief states because ‘belief’ is a folk-psychological notion and delusions do not fit the framework of folk-psychology by which agents interpret and predict one another (e.g., Murphy, 2012). Indeed, based on outdated philosophical accounts of belief, some psychologists have even ventured to define delusional reports as “empty speech acts”, suggesting that the content of delusions gives us no information about the person making the report or the world around them (e.g., Berrios, 1991).

In this paper, we challenge such conceptions of clinical delusions. We argue that delusions can be meaningful for the people making delusional reports, because delusions often make sense of people’s experiences. Delusions are also meaningful for interpreters engaging with the delusional reports, because delusions may well contribute to explanations and predictions of the speakers’ behaviour in context. Further, we make a case for the view that a more comprehensive and balanced analysis of delusions, not just as beliefs but also as ways of reconceptualising reality and the self, can be relevant to the question about what makes life meaningful.

In section one, drawing on the philosophical literature informed by cognitive psychology, our focus will be on how delusions can be considered meaningful and even adaptive in spite of representing significant failures of rationality and self-knowledge. In section two, drawing on the phenomenological literature, we explore the compensating and transformative power of delusions in the context of major disruptions affecting the basic relationship between the self and the lived world.

## A psychological perspective on delusions and meaning

When cognitive psychiatrists study behavioural anomalies, they share the conviction that a better understanding of what causes them can also shed light on the mechanisms underlying typical cognitive functioning. When the processes examined are those that culminate with the fixation of beliefs, it is widely recognised that cognitive biases and motivational factors can make a significant contribution and that some beliefs have the contents they do because of the operation of reasoning biases or self-serving biases.

The literature on delusions is an example of the general strategy that characterises cognitive psychiatry. The investigation into what makes delusions pathological turns into a broader exploration of the best theory of belief formation among competing accounts. In one influential version of the two-factor theory (Coltheart et al., 2010, 2011), the delusion is a pathological belief whose formation is due to two deficits: a neuropsychological impairment giving rise to anomalous data, and a cognitive dysfunction affecting the mechanisms responsible for belief evaluation. In a popular version of the predictive coding theory (Corlett, 2018), the delusion is a biologically adaptive belief whose formation enables the person to resume processes of automated learning that were disrupted by the incorrect signalling of prediction errors. Which theory is the most convincing account of delusions, or whether the theories can be fruitfully combined, are questions of great interest to philosophers but they will not occupy us here. Our purpose, instead, is to see what we can learn from the delusion formation literature about whether delusions are meaningful. Can delusions have meaning notwithstanding their epistemic irrationality and their often implausible, occasionally bizarre, content? We discuss cases in which the adoption of the delusion can be seen as a means to explaining the surrounding world and motivating people to engage with it.

### What are delusions?

Here are two first-person accounts of delusional experience and delusional beliefs:

I was driving home from work one day when I begin to hear voices inside of my head. It wasn’t just one voice talking—it was many in a low tone. I couldn’t distinguish what they were saying. It sounded like a radio between stations, with a lot of static. Time went by, and the voices became clearer. I was still very paranoid and delusional. *I thought people on my job were all judging me to see how well I did my job. I felt they could read what I was thinking and that I could talk to them without opening my mouth.* (Ruoss, 2019, our emphasis)

One night I thought that I had to stand up against the devil. I did not sleep and stood still in my room during the whole night. I thought about love and peace and concentrated on it in order to “send” love and peace via my thoughts to the outside world. When I saw the first rays of sunlight peeping through the window, I realized that the challenge was over. However, *Ramona, the representative of Mars, was angry with me since I had conquered the devil. The devil was an energy supplier to Mars. So now I thought Ramona wanted to kill me.* I thought she entered my body, and she was very strong. I thought my son and Christ tried to protect me. (Meijer, 2017, our emphasis)

In the powerful extracts above, people reflect on their past delusional experiences after reaching a stage where they manage their mental health more effectively. Reading the accounts, we get a sense of how delusional beliefs, like the ones we have emphasised in bold, are interconnected with the person’s emotions, perceptions, and other beliefs. The delusions are sometimes mundane in content (as in “People were all judging me”) and sometimes wildly implausible and idiosyncratic (as in “The devil was an energy supplier to Mars”).

In the *Diagnostic and Statistical Manual of Mental Disorders* (DSM-5), delusion is defined as:

A false belief based on incorrect inference about external reality that is firmly held despite what almost everyone else believes and despite what constitutes incontrovertible and obvious proof or evidence to the contrary. The belief is not ordinarily accepted by other members of the person's culture or subculture (i.e., it is not an article of religious faith). When a false belief involves a value judgment, it is regarded as a delusion only when the judgment is so extreme as to defy credibility (American Psychiatric Association, 2013, p. 819).

In cognitive psychology and psychiatry, delusions are regarded as beliefs that present three main epistemic features: they are held with great conviction (they are “firmly held”), they lack evidential support (they are “based on incorrect inference about external reality”), and are resistant to counterevidence (they are maintained “despite what almost everyone else believes and despite what constitutes incontrovertible and obvious proof or evidence to the contrary”). These three features are not dissimilar from what Jaspers identified as key featured of delusions: certitude, falsity/implausibility of content, and incorrigibility (Jaspers, 1963). However, in contemporary definitions of delusions, falsity or implausibility of content is often deemed a typical but not-necessary condition for delusions. Rather, what is thought to be distinctive about the delusion is the relationship between its content and the evidence available to the person at the time of adopting the delusion (Coltheart, 2007). What makes the utterance “People are all judging me” a delusion is not its implausibility (that content could as well be true) but the fact that the person reporting it does not seem to have sufficient evidence for it, at least not evidence that is intersubjectively available.

In the clinical context, delusions can have an *unusual* content, in the sense that they are idiosyncratic to the person reporting it and very unlikely to be shared by the other members of the person’s social groups (“The belief is not ordinarily accepted by other members of the person’s culture or subculture”). Although people in the same religious community may share a belief, say, in an all-powerful God, the person with religious delusions will have beliefs that other people from the same community reject, such as the belief that God assigned to her a special mission or that she “conquered the devil”.

It is widely accepted in the literature that delusions are an undesirable phenomenon—this is obvious from first-person reports where the ‘revelation’ about the world or the self that comes with the delusional belief is fraught with anxiety, doubt, fear, sense of danger. In some accounts, very unpleasant emotional reactions and erratic behaviour accompany the delusion, causing the person to distance themselves from family and friends, leave their studies, give up their jobs, and so on. That explains why in other influential definition of delusions, the fact that one experiences disruption in good functioning is regarded as a necessary condition for the person to qualify as “deluded”:

A person is deluded when they have come to hold a particular belief with a degree of firmness that is both utterly unwarranted by the evidence at hand, and that jeopardises their day-to-day functioning. (McKay et al., 2005, p. 315)

Not only are delusions thought to be irrational and disruptive, but they are also characterised as harmful. Indeed, Jerome Wakefield’s account of disorder as harmful dysfunction (Wakefield, 2007) has been applied to delusions, and delusional beliefs have been described as “harmful malfunctioning beliefs” (Miyazono, 2015). While this view of delusions as irrational, disruptive, and harmful dominates current psychopathological thinking, recently there has been a return to appreciating the psychological role that delusions may play in people’s attempts to make sense of their lives (McKay et al., 2005, 2007). As we shall see in Sect. 2, phenomenological as well as psychodynamic approaches have often highlighted the adaptive and potentially desirable side of delusional phenomena. As Jaspers said, delusion may bring about relief “from some enormous burden” (Jaspers, 1963, p. 98).

What are the prospects, then, for delusions to be meaningful and to enhance the sense that one’s life is meaningful? Is it possible to think of beliefs that are both irrational, disruptive, and harmful as having meaning and enhancing meaningfulness?

### Are delusions comprehensible?

It is worth reflecting on the fact that beliefs sharing the same epistemic features as those detailed in the DSM definition of delusion can be described as empowering in some circumstances. Let us consider the stories of Simon (Fulford & Jackson, 1997; Stanghellini et al., 2013) and Harry (Ritunnano et al., 2021):

Simon was a black, middle-class professional in his forties. He reported a series of “revelation” experiences conveyed by delusional perceptions and thought insertion. Nosographically, Simon’s experiences, if assumed to be pathological, might suggest a diagnosis of schizophrenia. However, from Simon’s perspective, his experiences were spiritual revelations: and consistently with this they were entirely beneficial to his life. His experiences and beliefs, whilst unusual in form and content, essentially enhanced his ability to function effectively: he won a difficult case thus advancing his career as a lawyer. Framing his experiences positively rather than negatively he avoided contact with doctors and instead integrated the information he (somehow) took from them in fighting and winning his court case.

(Stanghellini et al., 2013, pp. 291–292 abridged)

Mr Harry is a 33-year-old gentleman who has been complaining of being the target of a worldwide conspiracy for the past 5 years. […].When asked further about the challenges of conducting a life under the control of others, Harry replied: ‘If I went out one day and I realised that people weren’t expecting me to be there, it would be a real shock again . . .I would be...I don’t know...?! I got so used to people expect- ing me to be there and lash out with them...I would feel alone again, which is what everyone else feels, like alone. So people are like a family for me, it’s like a safety blanket, they make me feel so comfortable now...If I found out that they are not watching me and reading my mind, I would feel alone and crazy like everyone else. To feel like I have everyone following me around, whether it’s negative or positive, that alone is a force of power...knowing that you can influence people’s minds in the right way, I feel like Jesus (of course I’m not) but why not believe?’ (Ritunnano et al., 2021, epub ahead of print p. 2 abridged)

For Simon and Harry, the delusional experience is not lived as a burden or perceived as generating anxiety, but is seen as empowering and enlightening, at least for the most part. For Harry, the delusions seem to confer a sense of belonging and connection to others. For Simon, the delusions have a connection to his spiritual beliefs. And yet the beliefs Harry and Simon report are most implausible and also idiosyncratic to them.

A well-balanced account of delusions can explain the harm that delusions typically cause without ignoring the fact that in some circumstances, and often temporarily, delusions can have some benefits. The first step to arrive at such an account is to recognise that delusions are not “glitches in the brain” that evade a folk-psychological explanation, but outputs of a belief formation system whose primary aim is to make sense of the world. The second step is to acknowledge that delusions in schizophrenia have been conceptualised as explanations of unusual experiences and manifestations of the strong inclination of human agents to avoid uncertainty.

One obvious obstacle to seeing delusions as meaningful is the view that delusions represent a qualitatively different form of irrationality from other beliefs, a radical form of irrationality that prevents delusions from being understood or integrated in the folk-psychological framework that agents use to interpret and predict each other. However, as has already been argued (e.g., Bortolotti, 2018), the irrationality of delusions is not different in kind from the irrationality of many beliefs that we would characterise as neither delusional nor pathological, such as self-enhancing beliefs, prejudiced beliefs, and superstitious beliefs. As we saw in the previous section, delusions are held with conviction, typically badly supported by existing evidence when they are adopted, and resistant to counterevidence that emerges after their adoption. Similar to delusions, prejudiced beliefs are also firmly held, badly supported by existing evidence, and resistant to counterevidence: for instance, one may believe that another person belonging to a certain group has negative characteristics commonly associated with that group even when the behaviour of the person does not offer any evidence for the belief. This explains why a teacher may find it hard to believe that girls are good at maths or that a black child involved in a fight was not the one who started it. Similar to delusions, optimistically biased beliefs can be badly supported by the existing evidence and resistant to counter-evidence: for instance, one revises promptly a previous belief about having performed poorly on the basis of new evidence, but dismisses new evidence suggesting that one’s performance was worse than previously believed (Jefferson et al., 2017). The upshot of any thorough investigation into irrational beliefs is that it turns out to be very challenging to distinguish delusional from non-delusional beliefs, or pathological from non-pathological beliefs, on the basis of claims concerning the belief’s epistemic irrationality (Bortolotti, 2018).

Another obstacle to viewing delusions as meaningful comes from their unusual content: for some delusional reports, the impression is that the content of the belief is something that *could not* have happened, that is not just *implausible* but *impossible*. However, information about the person’s life—for instance, specific information about the experiences preceding the onset of the delusion—may help an interpreter understand why the reported belief has the content it does, even if it does not turn an irrational or implausible belief into a rational or a plausible one. This is true in particular for what Jaspers (1963) calls “delusion-like ideas”, and which differ from “primary” or “true” delusions exactly for this reason: because they are psychologically reducible to prior mental or affective states. Delusion-like ideas, Jaspers writes, emerge “*understandably* from preceding affects, from shattering, mortifying, guilt-provoking or other such experiences” (p. 96). In contrast, true delusions are defined as such precisely because they are psychologically irreducible, thus imposing a limit to what can be understood through mundane forms of empathy (Henriksen, 2013; Ratcliffe, 2012).

In a recent study (Gunn & Bortolotti, 2018), four people with delusions were interviewed, and the interview focused on the person’s lived experience, and on the events considered by the person to be significant prior to the onset of the delusion. It was found that many delusions seemed to play an almost *protective* function, enabling the person to cope with adversities affecting their social lives and their self-esteem. In the case of Barbara, she started believing that God was communicating with her by telepathic messages because she was his child and she was good: “as God was talking to me he was making sure that I knew there was nothing wrong with me. And he’s always there, whether I’m right, whether I’m wr… well, he, he says I’m never wrong, God says I’m never wrong.”

The delusion was formed after Barbara had heard voices for some time, unable to explain whose voices those were and what they wanted from her. Barbara’s delusional belief may be considered as an explanation for her unusual auditory experiences; furthermore, Barbara’s belief that she was special and that God was supporting her followed a very difficult time in her life, when her unfaithful husband had left her permanently and she was feeling both vulnerable and guilty about earlier decisions she made in her life. It is possible that (at least at the time when it was formed) the delusion might have protected Barbara from negative feelings about herself and prevented a suicidal attempt which was on her mind.

### Are delusions a response to adverse circumstances?

In some cases, it has been argued that delusions are not just meaningful, but they can confer meaningfulness to one’s life, and adopting a delusions can even be beneficial—often just in one respect and in the short term. In this section, we are interested in examining this counterintuitive claim. There are at least two ways in which delusions can be thought to confer meaningfulness: delusions emerging in the context of schizophrenia can help the person make sense of unusual experiences that would otherwise seem inexplicable and cause uncertainty and anxiety; and delusions emerging as a response to trauma or adversities can be conceived as protective responses to disruptive life events, making the person’s experience more bearable and especially providing *a sense of purpose* that helps keep depression at bay. The case of Barbara we analysed earlier is a good illustration of both claims: Barbara’s delusion that God is talking to her and her alone because she is special makes sense of the apparent fact that she is hearing voices that nobody else is hearing. At the same time, Barbara’s belief in God’s encouragement and support prevents her from being overwhelmed by the negativity of her recent experiences and from low self-esteem and desperation.

But what are the bases for thinking that delusions can confer a sense of meaningfulness? Following on the work by Jaspers and Conrad which we shall discuss in the next section, Glenn Roberts argues that delusion formation allows agents to attribute meaning to experience:

Delusion formation can be seen as an adaptive process of attributing meaning to experience through which order and security are gained, the novel experience is incorporated within the patient's conceptual framework, and the occult potential of its unknownness is defused […] Lansky […] speaks for many in asserting that ‘Delusion is restitutive, ameliorating anxieties by altering the construction of reality’. (Roberts, 1992, pp. 304–305 abridged)

In a study by Roberts (1991), patients with elaborated delusions score higher than patients in remission, rehabilitation nurses, and Anglican ordinands in the ‘purpose in life’ test and the ‘life regard’ index. The purpose in life test and the life regard index are both widely used and regarded as reliable means for measuring important aspects of the sense of meaning and purpose in people’s lives. Roberts concludes that ‘for some there may be satisfaction in psychosis and that [delusion formation] is adaptive’ (p. 19). As we saw, delusions explain the person’s puzzling experiences and, depending on their content, can also play a protective function, embellishing an unpleasant reality:

Both the specific contents of delusional beliefs and the experience of having found a powerful and comprehensive explanation, accompanied by a conviction of having discovered the truth, could be preferable to confronting reality again. In these circumstances there would be a movement towards elaboration and chronicity. Thus, discrepancies between delusional and real perspectives are likely to be resolved by further elaboration of delusion and adjustment of life circumstances in order to protect the beliefs from confrontation. A number of theorists with different perspectives have suggested that elaborate delusional systems may, in part, be perpetuated and mediated by the associated psychological benefits. (Roberts, 1992, p. 305)

In another study (Bergstein et al., 2008), it was found that elaborated delusions contribute positively to the ‘sense of coherence’: indeed, the sense that one’s life is meaningful is greater in a population with elaborated delusions than in a non-clinical population. The sense of coherence is defined as ‘a global orientation that expresses the extent to which one has a pervasive, enduring though dynamic, feeling of confidence that (1) the stimuli deriving from one's internal and external environments are structured, predictable, and explicable; (2) the resources are available to one to meet the demands posed by these stimuli; and (3) these demands are challenges, worthy of investment and engagement’ (Antonovsky, 1987, p. 91).

Both sense of coherence and meaningfulness correlate with wellbeing but what is striking in the way in which purpose in life, life regard, and sense of coherence are conceptualised and measured is that they capture what it is for someone to have a sense of agency, the capacity to intervene on their physical and social environment, and to maintain the motivation to do so. And sense of agency and motivation seem to be key components of what it takes for people to find their lives meaningful, and cultivate an interest in attaining goals that they find worth pursuing.

## Delusion and lived experience

As a philosophy *of meaning* (Crowell, 2001), phenomenology is intended here as an enterprise where the distinction between the meaning of the world and its being is not a straightforward one (Zahavi, 2017). Although our investigation is not concerned with metaphysical issues about the constitution of reality, delusion—often characterised as a misrepresentation of basic observational facts about the world—calls into question the common-sense understanding of everyday life. By bracketing our assumptions about what is ordinarily taken to be the primary source of meaning, phenomenology allows us to take a fresh, unprejudiced look at the lived experience of delusion to see if we can make sense of it. Momentarily setting aside “the good, the true, and the beautiful” as the greatest sources of meaning in life (Metz, 2011), a critical engagement with the phenomenological method allows us to discover hidden meaningfulness within the irrational, the harmful, and the incomprehensible.

A long tradition of phenomenological psychopathology has attempted to rescue the ‘voice of madness’ from a priori attributions of meaninglessness by gaining access to the first-person perspective. Indeed, most phenomenologists would agree that “it is impossible to define delusion without being concerned with the patient’s experience” (Parnas, 2004, p. 151). This perspective opens up a world of new meanings where something more ‘fundamental’ about human existence is revealed (Sass, 2019).

Following this lead, we draw on phenomenological insights to support the claim that delusions are not just the result of applying idiosyncratic ‘framework propositions’ to one’s experience of reality (Campbell, 2001) but arise in the context of a global transformation of the ontological framework of experience (for a comprehensive critical overview, see Feyaerts et al., 2021). Next, we go on to show how delusions can make sense and give meaning to our experiences, in the context of a general disintegration of previously taken-for-granted meaning patterns. Drawing on the influential book *Madness and Modernism* by Sass (2017), we argue that the very alienating character of some delusional states, along with an intense self-awareness and rejection of common-sense conventions, can enhance meaning in life by opening up new possibilities for creative expression.

### What is it like to experience a delusion?

In his most important and foundational contribution to the field of psychopathology, Jaspers (1963) is clear on the fact that criteria of certitude, falsity/implausibility, and incorreggibility merely point to external characteristics of delusions—which do not take us very far in our inquiry into their psychological nature or meaning. If we want to move beyond these mere external features, Jaspers writes, we need to confront the question: “what is the primary experience traceable to the illness and what in the formulation of the judgment is secondary and understandable in terms of that experience?” (p. 96). Only by addressing this question, we may identify what is phenomenologically peculiar about delusional experience, or in other words, what makes an experience delusional in the first place. This is no simple task for the phenomenologist, who is soon confronted with “quite alien modes of experience [which] remain largely incomprehensible, unreal and beyond our understanding” (p. 98, abridged). But this should not be a reason to stop *trying* to understand. Indeed, there is much that can be learned by way of getting closer to the primary experience itself: “we find that there arise in the patient certain primary sensations, vital feelings, moods, awarenesses” (p. 98). In the context of primary delusions (more commonly found in schizophrenia) such feelings and sensations form a well characterised and distinct clinical phenomenon called ‘delusional atmosphere’ or ‘delusional mood’—which remains however extremely difficult to describe due to the very strangeness and ineffability of the experience itself. Consider for example the following first-person account, proceeding by metaphor:

Objects are stage trappings, placed here and there, geometric cubes without meaning. People turn weirdly about, they make gestures, movements without sense; they are phantoms whirling on an infinite plain, crushed by the pitiless electric light. And I - I am lost in it, isolated, cold, stripped purposeless under the light. A wall of brass separates me from everybody and everything. In the midst of desolation, in indescribable distress, in absolute solitude, I am terrifyingly alone; no one comes to help me. This was it; this was madness […] Madness was finding oneself permanently in an all embracing Unreality. I called it the “Land of Light” because of the brilliant illumination, dazzling, astral, cold, and the state of extreme tension in which everything was, including myself. (Sechehaye, 1970, p. 33)

Jaspers describes it as follows:

Patients feel uncanny and that there is something suspicious afoot. Everything gets *a new meaning*. The environment is somehow different—not to a gross degree—perception is unaltered in itself but there is some change which envelops everything with a subtle, pervasive and strangely uncertain light. A living-room which formerly was felt as neutral or friendly now becomes dominated by some indefinable atmosphere. Something seems in the air which the patient cannot account for, a distrustful, uncomfortable, uncanny tension invades him. (Jaspers, 1963, p. 98; emphasis original)

People often struggle to communicate their puzzling experience through language and some describe living in a ‘real simulation’ or a ‘fake reality’ reminiscent of the movie *The Truman Show.* In these moments, other people may look like mannikins, puppets or robots wearing a mask, or they may appear two-dimensional as if they were artificially projected on the backdrop of a theatrical scenery (Sass, 2017, p. 29). Accounts from the phenomenological literature show the destabilisation of previously taken-for-granted meanings, whereby people with delusions feel completely lost outside of the pre-reflective and normative matrix of accepted meanings (Pienkos et al., 2017). For example:

I guess it is mostly like, that’s what I can describe it, as a dream, but it’s also like is this actually my life, is this actually what I perceive it to be, or am I actually like, that big philosophical thing that you see in movies, if you zoom out, is this actually the universe, or just some kind of an amoeba in a petri dish in some kind of larger universe. (Pienkos et al., 2017, p. 198)

Life seems to have lost its reassuring sense of reality and has taken on the precariousness of a lucid dream. Most often the delusional atmosphere is fraught with anxiety, disquietude and anguish, but the ‘lucid dream’ can be perceived in some cases as an exciting and illuminating experience. There is an increasing tension coupled with an unbearable sense of ambiguity and uncertainty about the future. Objects seem to be floating free from their background (Matussek, 1987), disconnected from their habitual meaning frameworks while the everyday world is undergoing some sort of inexplicable metamorphosis. This in turn triggers an exaggerated and morbid hyper-rationality and introspective activity, whereby people with delusions seem to gain access to certain ontological facets of human life that remain usually unattended and too often neglected (Sass, 2019).

This is reminiscent of Heidegger’s experience of Angst*.* For Heidegger (2007), Angst is not a meaningless phenomenon (even if in it we are not able to carry out our everyday life), rather it provides a special access to the ontological (Withy, 2015, pp. 77–92). Something *very real* about the human condition is revealed through Angst, namely the possibility of not being oneself. Having acknowledged our perpetual “fall” into everydayness and inauthenticity, the possibility of becoming who we really are (authenticity) is opened up; we are called upon to be ourselves. Alice Holzhey-Kunz (2020) refers to Angst as a fundamental philosophical experience that tells us an unfathomable truth about human life, which is necessarily forgotten by normal people when attending the demands of everyday life. This truth entails the burdensome awareness that our life is fundamentally dominated by the law of time and its finitude cannot be escaped. This raises the question: can delusional forms of existence unveil something about the meaning of life?

### Delusions as discovery and revelation

In the section above, we have referred to some phenomenological accounts of the perceptual alterations that characterise pre-delusional states, wherein the very sense of reality seems to go awry and the agent is lost in a permanent state of “something is going on; do tell me what on earth is going on” (Jaspers, 1963, p. 98). The German psychiatrist Klaus Conrad, in his seminal work on the formation of schizophrenic delusion (Conrad, 1958), calls this initial phase “Trema”, emphasising the expectational and suspenseful character of the experience—similar to the actor’s “state of tension” (“Spannungszustand”) before going on stage. In Conrad’s model, the “trema” phase is often followed by or intertwined with delusional mood and includes a number of different emotional, affective and atmospheric features such as an increased basic affective tone, mistrust and depressive-like mental states such as guilt, anxiety, and fear of death (Henriksen & Parnas, 2019, pp. 747–750). Growing out of the “trema”, the delusional mood becomes increasingly self-referential; the neutrality of the experiential background is lost and whatever is happening or about to happpen is directed against the subject. This stage may progress into Apophany (Greek apo [away from] + phaenein [to show]), wherein the delusional meaning is experienced as a revelation or “Aha-Erlebnis” which alleviates the previous unbearable sense of impending doom. In the apophanic stage, one promptly makes sense of what was previously only alluded to and is struck by a revelation. This opens up a new, hidden meaning intended especially for that person.

Jaspers similarly describes the sudden formation of delusional ideas following delusional atmosphere, as in this example from the writings of a patient:

It suddenly occurred to me one night, quite naturally, self-evidently but insistently, that Miss L. was probably the cause of all the terrible things through which I have had to go these last few years (telepathic influences, etc.). I can’t of course stand by all that I have written here, but if you examine it fairly you will see there is very little reflection about it; rather everything thrust itself on me, suddenly, and totally unexpected, though quite naturally. I felt as if scales had fallen from my eyes and I saw why life had been precisely as it was through these last years…(Jaspers, 1963, p. 103)

Jaspers recognises the soothing effect provided by the experience of finding “a fixed point” to cling on:

this general delusional atmosphere with its vagueness of content must be unbearable. Patients obviously suffer terribly under it and to reach some definite idea at last is like being relieved from some enormous burden […] the achievement of this brings strength and comfort, and it is brought about only by forming an idea, as happens with health people in analogous circumstances (Jaspers, 1963, p. 98).

Here the emergence of the belief out of the delusional atmosphere appears as something meaningful, possibly also warranted and necessary to resolve a situation of indefinite anticipation. Many would indeed agree that searching for meaning is a fundamental human need in the context of any life event, and this applies to a greater extent when we are confronted with ambiguity and uncertainty. Some authors (Maher & Ross, 1984) have suggested that the person engages in a process of empirical observations and hypothesis testing, which is not dissimilar from that of the researcher trying to unravel a scientific mystery. The long sought missing detail finally provides the explanatory insight that solves the enigma and dissipates anxiety, perplexity and confusion—thanks to the formation of a delusion. This is a sort of eureka moment when everything falls into place and a new *understanding* of reality is established, which brings forth a sense of relief. Framed in this way, the newly developed delusional framework can be viewed as meaningful and adaptive insofar as agents are relieved of negative feelings and acquire the necessary hermeneutical, affective and pragmatic resources to understand their world and invest in foreseeable challenges. While bringing forth a sense of relief and new affordances, however, this shift in perspective may also undermine habitual and trusted views of oneself, others and the world, fuelling what Sips calls a dialectic of aha- and anti-aha experiences (Sips, 2019; Van Duppen & Sips, 2018). The notion of anti-aha experience highlights the dynamic involvement of one’s personal and interpersonal contexts and narratives in making sense of delusional experiences and initiating problem finding, which in some cases may lead to transformative and spiritual growth (Nixon et al., 2010).

Sass and Pienkos (2013) have suggested other possible compensatory features of delusion, for example related to the wish of some people to escape a reality that is either intolerable, unsatisfying or unsafe. In these cases, the delusional reality may provide additional meaning in life, a sort of preferred reality where the agent is protected from unbearable suffering, pain, depression and in some cases suicidality such as in the case of Barbara discussed earlier (Gunn & Bortolotti, 2018).

The new meaningfulness unveiled through the delusional experience can take up different themes—for example persecutory, grandiose, religious, somatic and so on. Phenomenologists have suggested that some schizophrenic delusions are concerned with “ontological” themes about the metaphysical status of the universe (Parnas, 2004), rather than mundane or “ontic” affairs. Ke̜piński (1974) describes three main metaphysical taints that often colour schizophrenic delusions: ontological (e.g., about the nature of being), eschatological (e.g., about the end of the world, and charismatic (e.g., about the meaning of life). He says:

the patient is not inactive when the world is exposed to apocalyptic events. He is in the central position of that world. He may feel immortal, immaterial, almighty, as God or devil; the fate of the world depends upon him […] The world is threatened by annihilation, and the patient wants to warn mankind, offer himself for the sake of humanity […] *The meaning of his life reveals itself to the patient: a great mission, an act of heroism, martyrdom*. (transl. in Bovet & Parnas, 1993, pp. 121–122; our emphasis).

Therefore, through the development of delusion, life seems to gain a new meaning where the person often feels superior, exceptional, and closer to the truth. The poet Gerard de Nerval, in his illness memoir *Aurelia*, rejoices while recounting the things he has seen as a spirit: “how happy I was in my new-found conviction! Those lingering doubts about the immortality of the soul which beset even the best of minds were now laid to rest. No more death, no more sorrow, no more anxiety” (Nerval, 1999, p. 277).

Considering the sense of enlightenment and truthful perfection that pervades these delusional worlds, it would seem counterintuitive to dismiss such instances as senseless speech, devoid of any meaning. However, it would be misleading to think that all delusions are accompanied by joyful feelings and experienced with a positive outlook. More often than not, in the context of severe mental disorders such as schizophrenia, delusions may bring about intense feelings of paranoia, fear, apprehension, anguish, guilt, shame, depression, or even annihilation. The very sense of existing as a unified, separate being might be affected (Parnas & Sass, 2001). People with delusions may report being constantly followed, laughed at, spied upon, or poisoned. Privacy and ego boundaries can be seriously damaged to the extent that “a schizophrenic may say that he is made of glass, of such transparency and fragility that a look directed at him splinters him to bits and penetrates straight through him” (Ratcliffe & Broome, 2012). Some people describe losing control over their own actions, as if an alien force were controlling their movements or their thoughts. Some believe that their thoughts have been implanted into their brain by an alien force and are being broadcasted across the world. The end of the world might be impending. In no ways can the person be reassured of the unlikelihood of such a catastrophic event: “everything is so dead certain that no amount of seeing to the contrary will make it doubtful” (Jaspers, 1963, p. 104).

In these cases, a new sense-making is established out of the delusional mood albeit one that leads to a world of persecution and isolation. Communication with the person might become difficult because there is no shared background of significance on which the intersubjective world can be co-constituted (Fuchs, 2020). The possibility of a shared reality is taken over by a solipsistic world-view, where the self is entrapped amid paradoxical feelings of centrality and self-dissolution (Parnas & Sass, 2001).

Can such an extreme existential position still be meaningful? A distinctive meaningfulness of solipsistic acts can certainly be claimed inasmuch as it provides a sort of adhesive, holding together the pieces of a shattered self (Humpston, 2018). This view is grounded on the idea that schizophrenic delusions originate on the background of pre-existing disturbances of the basic sense of self (or minimal self), as conceptualised in the ipseity disturbance model of schizophrenia (Sass et al., 2018). On this account, certain instances of bizarreness can be explained as the result of three interconnected aspects of self-disturbance: (1) diminished *self-presence*, referring to a reduced sense of existing as a living agent; (2) *disturbed grip or hold* on the world, that is a destabilisation of the meaning-structure, salience-pattern or reality status of the world; (3) *hyperreflexivity* in the sense of an exaggerated—mainly avolitional—reflective consciousness (Sass & Byrom, 2015). Hyperreflexivity leads to increasing objectivation of introspective experience whereby tacit and automatic phenomena become focal objects of awareness. This sort of detached hyperconsciousness need not be inherently pathological or uniquely present within schizophrenia. Affinities have been observed between certain kinds of anomalous self-experience and, for instance, states of intense introspection or meditation such as those sought after by modernist and post-modernist artists (for example within Surrealism and Russian Futurism art movements). In these contexts, a volitional kind of hyperconsciousness was considered a fundamental aesthetic practice aimed at suspending the conventional meaning attributions to unveil the concreteness and abstract particularity of external objects (Sass, 2017).

### Delusions and creativity

So far, we have come to appreciate the sense-making features of some delusions which arise in the context of a puzzling and uncanny pre-delusional state. On this account, delusions can be meaningful insofar as they afford existential rescue from the uncertainty of a shattered sense of presence. As a patient with schizophrenia puts it: “Delusions are an attempt to explain a very deep restlessness. It is an attempt to seek rescue in a story in which you eventually get lost” (Henriksen et al., 2010, p. 366). Through this process, a new narrative is created that re-establishes a sense of coherence, yet appears fundamentally disconnected from the shared world. The structuring of a new meaning, often characterised by a self-reflective and hyper-aware focus on theoretical and metaphysical aspects of experience, is coupled with a de-structuring of practical, ontic, or common-sense meanings that provide the experiential background for mutual understanding and sharing of social practices. Indeed, it is not surprising that “dissociality” (or “social dysfunction” in DSM terminology) is a fundamental feature of schizophrenia which is typically characterised as the lack of appropriate interpersonal skills, failure of social adjustment or withdrawal from social life (Stanghellini & Ballerini, 2011). In keeping with a “deficit” view of schizophrenia (i.e., it falls short of some standards of “goodness”), we often find delusion being related to a fundamental lack of or decline in certain end values of intellectual attainment, moral fulfilment, and aesthetic worth. While these are certainly promising candidates for our search into what makes a life meaningful (Metz, 2011), we believe that they are not the only places where meaningfulness can be found. For this *quale* might be concealed in certain unconventional modes of being in the world that have been traditionally associated with notions of emptiness, defect and insanity. One way in which the meaningfulness of certain unusual experiences might be accessed is through the analysis of modernist and postmodernist artwork.

In his book *Madness and Modernism*, Sass (2017) provides a compelling argument for the recovery of the voice of schizophrenia from the defective world of blindness, disease and meaninglessness, where it has been segregated for centuries by most Western ideologies. Sass contrasts the traditional portrait of madness—evoking darkness, demonic forces, and incomprehensible beastlike sounds (epitomised for example in the painting by Francisco Goya “The Madhouse at Saragossa”)—with that of immense, bright and timeless metaphysical landscapes (such as those represented in Giorgio De Chirico’s early canvases such as “Melancholy of a beautiful day” or “The enigma of a day”). Here there is no spontaneous and passionate expression of the primordial unity between the self and the world—as considered in post-romantic terms—but an all-encompassing and enigmatic sense of significance that refers to neither the self nor to the world but to the very act of consciousness. When the meaning of life—as conceived of in our everyday mundane dimension of living—disintegrates, what is it to be found under the familiar surface of reality? By affording deeper insights into the nature of existence, unusual experiences such as those arising in the context of the prototypical surrealist mood (as a sort of intense and detached introspection), have often been regarded as a mysterious source of artistic creativity. The parallel that Sass has been able to draw so cogently with the state of delusional mood remains however contentious in other pathographical readings of modern poetic and artistic work. Indeed, the beneficial impact of psychotic processes on meaningfulness can be difficult to defend, while simultaneously acknowledging the inescapable destructive effects of the illness over time.

Jaspers was fully aware of this psychological and existential conundrum when he wrote: “Just as a diseased oyster can cause the growth of pearls, by the same token schizophrenic processes can be the cause of mental creations of singular quality” (Jaspers, 1977, p. 134). In his pathographical analysis of Friedrich Hölderlin and Vincent van Gogh, Jaspers recognises the meaningful interplay between the extraordinarily talented personalities and their psychotic suffering. Referring to the acute onset of illness in Hölderlin, Jaspers describes a period filled with disintegrating forces and disciplining attitudes where the poet attempts to preserve a sense of coherence, order, and meaning in the face of other-worldly dangers and divine revelations (Jaspers, 1977, p. 146). Though clearly immersed in a delusional reality imbued with mythical actuality, Hölderlin’s “philosophy of life, formerly filled with longing, with conflict, suffering and remoteness, becomes during the period of the schizophrenic process more actual, more immediate, more fulfilled, elevated at the same time into a more general, objective, impersonal, timeless sphere” (Jaspers, 1977, p. 144).

Moreover, in his comparison between Hölderlin and van Gogh, Jaspers emphasises how both artists had a similar experience, following the first acute onset of psychosis—which he describes as follows:

“a preliminary state of philosophical turbulence, coupled with an increased feeling of security and of a nonchalant feeling of self-assertion, as well as a noticeable change in the nature of their works which are understood by then as well as others as growth and as the conquest of their goal” (Jaspers, 1977, p. 193).

It seems therefore fitting to defend a contribution of the delusional experience towards enhancing meaningfulness, though recognising the deleterious long-term effects of the schizophrenic condition, which was eventually evident in the final phase of both artistic lives. This is certainly not to say that suffering is necessary to make a life meaningful, but rather that—under certain conditions—delusional experiences can contribute to the sense that our lives are coherent, directed and worthy of investment. Exemplar cases of superlative meaningfulness, such as the lives of Hölderlin and van Gogh, further suggest that delusion can contribute towards the achievement of great significance. In these cases, however, the meaningfulness arising from the realisation of such superlative projects is critically intertwined with significant psychological costs and existential challenges connected with chronic illness.

## Conclusion

Delusions have often been portrayed as paradigmatic instances of meaninglessness and incomprehensibility. This view—more widespread since the so-called *operational turn* in the 1970s—has been shared across medicine, psychology and philosophy, and is widely held in popular culture; it has been reinforced by stigmatising media coverage of how mental health may be at the origin of inexplicable behaviour, as well as by works of fiction in literature and cinema where people with delusions are routinely represented as irrational, unpredictable, and dangerous. The fact that some delusions have unusual content, give rise to behaviour that is sometimes difficult to predict, and have undeniably harmful consequences for the person experiencing the delusions has contributed to the argument that delusions are both meaningless themselves, and strip meaning away from people’s lives.

But maybe there is more to delusions than the idea that the person is making an incorrect judgement about external reality. This narrow account of delusion fails to acknowledge both the person’s life story and some basic experiential changes that affect the person’s way of seeing the world: these may remain inaccessible to an external observer without adequate background knowledge.

By starting from the first-person perspective, we explored dimensions of delusion that are often out of focus due to their apparent incoherence with what is conventionally assumed to make a life meaningful–such as truth, beauty, and goodness. We have shown that it makes sense to consider delusions as meaningful, both from the perspectives of cognitive psychology and phenomenology. Delusions have a meaning for the person reporting them (in light of their experiential background) and for a suitably informed interpreter (who has the willingness to listen and the resources to understand).

We have also shown that, in some circumstances, adopting the delusion contributes to the person’s life having meaning and purpose, and at least temporarily and imperfectly restores an already compromised engagement with the person’s physical and social environment. Indeed, from a subjective point of view, the formation of a delusion can contribute to re-establishing a sense of coherence, directedness, and belonging—particularly in the context of previously distressing life events. Finally, we have briefly discussed cases in which—even from an objective point of view—people are able to attain superlative intellectual and creative achievements thanks to the transformative power of delusional experiences.

## Data Availability

Not applicable.
